# A randomized, double-blind, placebo-controlled study of losmapimod in patients with facioscapulohumeral muscular dystrophy: Results of the REACH study

**DOI:** 10.1177/22143602261419558

**Published:** 2026-02-06

**Authors:** Nicol C Voermans, Jeffrey M Statland, Lawrence J Hayward, Angela Rosenbohm, Adolfo López de Munain, Sabrina Sacconi, Doris G Leung, Umesh A Badrising, John Vissing, Benedikt Schoser, Nuria Muelas, Hanns Lochmüller, Enrico Bugiardini, Leo H Wang, Lorenzo Maggi, Thomas Ragole, Alan Pestronk, Johanna I Hamel, Namita A Goyal, Lawrence Korngut, Elie Naddaf, Amy Harper, Perry B Shieh, Cornelia Kornblum, Valeria Sansone, Angela Genge, Giorgio Tasca, John Jiang, Marie-Helene Jouvin, Rabi Tawil, on behalf of the REACH Investigators

**Affiliations:** 1 Department of Neurology, Donders Institute for Brain, Cognition and Behaviour, 6034 Radboud University Medical Center, Nijmegen, The Netherlands; 2 Department of Neurology, 21638 University of Kansas Medical Center, Lawrence, KS, USA; 3 Department of Neurology, 12262 University of Massachusetts Chan Medical School, Worcester, MA, USA; 4 Department of Neurology, Ulm University Hospital, Ulm, Germany; 5 Neurociences Research Centre, University Hospital Donostia, Donostia, San Sebastián, Spain; 6 Peripheral Nervous System & Muscle Department, Pasteur 2 Hospital, Nice University Hospital, Nice, France / Institute for Research on Cancer and Aging of Nice, CNRS, INSERM, 37045 Côte d’Azur University, Nice, France; 7 Center for Genetic Muscle Disorders, 20870 Kennedy Krieger Institute, Baltimore, MD, USA; 8 Department of Neurology, 4501 Leiden University Medical Center, Leiden, The Netherlands; 9 Copenhagen Neuromuscular Center, Rigshospitalet, 4321 University of Copenhagen, Copenhagen, Denmark; 10 Department of Neurology LMU Clinic, Friedrich-Baur-Institute, Munich, Germany; 11 Neuromuscular Diseases Unit, Neurology Department, Hospital Universitari i Politècnic La Fe, Valencia, Spain; 12 Division of Neurology, Department of Medicine, 113684 The Ottawa Hospital, Ottawa, Canada; 13 Queen Square Centre for Neuromuscular Diseases, 98546 National Hospital for Neurology and Neurosurgery, London, UK; 14 Biomedical Research Centre, National Institute for Health Research, University College London Hospitals, London, UK; 15 7284 Department of Neurology, University of Washington, Seattle, Washington, USA; 16 Neuroimmunology and Neuromuscular Diseases Unit, 9328 Fondazione IRCCS Istituto Neurologico Carlo Besta, Milan, Italy; 17 Department of Neurology, 12225 University of Colorado School of Medicine, Aurora, CO, USA; 18 Departments of Neurology and Pathology and Immunology, 12275 Washington University School of Medicine, Saint Louis, MO, USA; 19 Department of Neurology, 6923 University of Rochester Medical Center, Rochester, NY, USA; 20 Department of Neurology, 8788 University of California, Irvine, Orange, CA, USA; 21 Department of Clinical Neurosciences, Hotchkiss Brain Institute, 2129 University of Calgary, Calgary, Canada; 22 Department of Neurology, 6915 Mayo Clinic, Rochester, MN, USA; 23 Department of Neurology, Children's Hospital of Richmond at Virginia Commonwealth University, Richmond, VA, USA; 24 Departments of Neurology and Pediatrics, 8783 University of California Los Angeles, Los Angeles, CA, USA; 25 Department of Neuromuscular Diseases, Center for Neurology, 39062 University Hospital Bonn, Bonn, Germany; 26 The NEMO Center in Milan, Neurorehabilitation Unit, University of Milan, ASST Niguarda Hospital, Milan, Italy; 27 Neurology Clinic, The Neuro – Montreal Neurological Institute Hospital, Montreal, Canada; 28 John Walton Muscular Dystrophy Research Centre, Newcastle University and Newcastle Hospitals NHS Foundation Trusts, Newcastle Upon Tyne, UK; 29 461213 Fulcrum Therapeutics, Inc., Cambridge, MA, USA

**Keywords:** Facioscapulohumeral muscular dystrophy, skeletal muscle loss, reachable workspace, DUX4, quantitative dynamometry

## Abstract

**Background:**

Losmapimod is an orally administered small molecule and selective p38α/β mitogen-activated protein kinase (MAPK) inhibitor able to reduce aberrant expression of *DUX4 in vitro* and thereby potentially slowing disease progression in patients with facioscapulohumeral muscular dystrophy (FSHD).

**Objective:**

This global, randomized, placebo-controlled, double-blind phase 3 study in patients with FSHD1 and FSHD2 examined the efficacy and safety of losmapimod over a 48-week treatment period compared to placebo (NCT05397470, EUDRACT 2022-000389-16).

**Methods:**

The primary endpoint was change in quantification of reachable workspace (RWS) expressed as relative surface area (RSA). Other endpoints included measures of muscle composition (fat content and lean muscle) using magnetic resonance imaging (MRI), muscle strength using quantitative dynamometry, and quality of life measures.

**Results:**

130 participants received losmapimod and 130 participants received placebo, with 252 participants completing the 48-week treatment period. There were no statistically significant differences between groups in change in RSA and all secondary efficacy endpoints from baseline to Week 48. Losmapimod treatment was well-tolerated, and most adverse events were mild.

**Conclusions:**

Losmapimod was generally well tolerated with a favorable safety profile at a dose of 15 mg twice daily. Although none of the efficacy endpoints were met, study design and data from the study may inform future studies of FSHD therapies.

## Introduction

Facioscapulohumeral muscular dystrophy (FSHD) is a rare and progressively disabling genetic muscle disorder that can lead to significant physical impairment and disability. FSHD is one of the most common muscular dystrophies with an estimated prevalence of 4 to 12 out of 100,000 in Europe and North America.^1–3^ FSHD symptoms can develop at any age from infancy to late adulthood but are often first detected in the second decade of life.^4^ Two genetically distinct forms of FSHD (FSHD1 and FSHD2) are caused by aberrant expression of the transcription factor DUX4 in skeletal muscle. Approximately 95% of patients have FSHD1, which is due to a microsatellite repeat contraction in the D4Z4 region on chromosome 4q35 and is inherited in an autosomal dominant fashion. The remaining 5% of patients have FSHD2, which is due to the presence of pathogenic variants in D4Z4 epigenetic repressors in the absence of large D4Z4 repeat deletions and whose transmission is not well understood.^5,6^ In both FSHD1 and FSHD2, derepression of *DUX4* in skeletal muscle cells *in vitro* leads to the activation of pathological cell processes including expression of cytokines, cellular stress, and programmed cell death.^7–10^

The clinical manifestations are highly variable, consisting of muscle weakness and atrophy affecting predominantly the face, periscapular, and abdominal regions, as well as lower limbs in most patients, often in an asymmetrical fashion. Muscle strength decreases at a rate of approximately 1% to 4% per year, and the 6-year risk of wheelchair use is approximately 24%.^6^ Muscle biopsies from patients with FSHD show, nonspecific myopathic changes with variable degrees of inflammatory infiltrates, fibrosis, and fatty replacement.^4,11,12–14^ In a survey of FSHD patients, the most cited symptoms cause difficulty in daily life were general muscle weakness, difficulty using arms or hands, not being able to walk, fatigue, or impaired mobility, energy, and endurance.^15^ Symptomatic treatment, including low-intensity aerobic exercise and occupational therapy focused on energy self-management may help.^4,16^ However, no approved drugs address the pathophysiology of FSHD.

Losmapimod, an orally administered small molecule and selective p38α/β mitogen-activated protein kinase (MAPK) inhibitor that reduces stress-related gene expression, including that of *DUX4*, was originally developed by GSK for numerous non-FSHD indications with pro-inflammatory pathogeneses. While losmapimod was not effective for these indications, these clinical studies with losmapimod provided robust evidence of safety in over 3500 patients. Independently, p38α/β MAPK was shown to be a key regulator of *DUX4* expression, leading to the hypothesis that losmapimod could be a candidate therapy targeting the pathophysiology of FSHD. In preclinical models of FSHD, losmapimod decreased *DUX4* expression in differentiating FSHD patient-derived myotubes without negatively impacting myogenesis.^7,17,18^

The design of the current clinical study was based on several prior studies. Based on the results of a phase 1 study conducted in healthy volunteers and participants with FSHD, a losmapimod dose of 15 mg twice daily was selected.^12^ Other studies led to the selection of the following efficacy assessments: reachable workspace (RWS), dynamometry, and whole-body musculoskeletal MRI.^12,19,20^ RWS measures upper extremity function and range of motion and can capture change over time that correlates with function required for activities of daily living.^21–27^ Disease severity was also evaluated using the Ricci clinical severity score, that grades extent of muscle weakness from facial to low-limb regions.^28^ Evaluation of the upper extremity is of particular importance in FSHD because the facial and scapular stabilizer muscles are often involved early in the disease process.^29^ RWS is quantified as the relative surface area (RSA), which represents the area of a hemisphere centered on the shoulder joint in which the arm can move relative to the torso and is derived from the 3D motion capture system of RWS (using a Kinect sensor). Lower limb mobility assessments such as classic and FSHD-optimized Timed Up and Go (TUG), dynamometry, quantitative whole-body musculoskeletal MRI (which assesses muscle-fat content expressed as muscle fat infiltration (MFI))^12,30^ were also evaluated as potentially useful clinical outcome assessments. The classic and FSHD-optimized TUGdid not however show sensitivity to change over time in participants with FSHD and were not included for evaluation in this study.

A phase 2, randomized, double-blind, placebo-controlled clinical study of losmapimod was conducted in 80 patients with FSHD1 (ReDUX4).^20^ The primary endpoint, change in DUX4-driven gene expression in muscle biopsies performed in STIR + muscles, showed no differences from baseline in the losmapimod and placebo groups and no difference between groups. However, losmapimod was associated with some improvements in imaging and functional outcomes. including improvement or stabilization of RWS in dominant and non-dominant arms and improvement in Patient Global Impression of Change (PGIC) reports. Collectively, these data led to the selection of RWS as the primary outcome measure in the phase 3 study.

Here we report the results of the REACH clinical study, a phase 3, randomized, double-blind, placebo-controlled study designed to assess the efficacy and safety of losmapimod for treating people with FSHD1 or FSHD2. Although the REACH study did not meet its primary endpoint, the study design and data from the study are presented here to report the findings since they are of great interest for the FSHD community and may be useful for future studies.

## Patients and methods

### Study participants

Males and females with ages 18 to 65 years, inclusive, with a diagnosis of FSHD1 or FSHD2 confirmed by genetic testing^5^; a Ricci clinical severity score of 2 to 4 (5-grade scale); and a RWS total RSA score (Q1-Q4; range 0–1) without weight in the dominant upper extremity of at least 0.2 and no more than 0.7 were enrolled. Enrolled participants did not have contraindications to MRI and did not have prior or current medical conditions that would impair study completion or interpretation of results. Complete inclusion and exclusion criteria are provided in Supplemental Table 1.

Medications that may affect muscle function (e.g., statins, steroids, testosterone or other growth hormone agonists, beta agonists, creatine, colchicine, benzylpenicillin) were allowed, provided the participant was on a stable dose for at least 3 months prior to the first dose of study drug and continued the stable dose for the duration of the study. Written informed consent was obtained prior to study participation. The study was approved by the independent ethics committee/institutional review board at each clinical site and was conducted in accordance with the Declaration of Helsinki and International Council on Harmonisation (ICH) Good Clinical Practice guidelines.

### Study design

REACH was conducted at 33 sites in 9 countries (USA, Canada, Great Britain, France, Spain, Germany, Italy, the Netherlands, and Denmark) between June 2022 and August 2024 (NCT05397470). Following a screening period of up to 42 days, enrolled participants were randomly assigned 1:1 to receive either losmapimod 15 mg or matched placebo capsules twice daily for 48 weeks. Losmapimod and placebo were administered in double-blind fashion. Study drug was orally administered with food. Following the placebo-controlled treatment period, participants had the option to rollover to an open-label extension, which has since been terminated. Randomization was stratified by FSHD type and repeat number category (FSHD1, 1–3 repeats; FSHD1, 4–9 repeats; and FSHD2).

**Figure 1.**
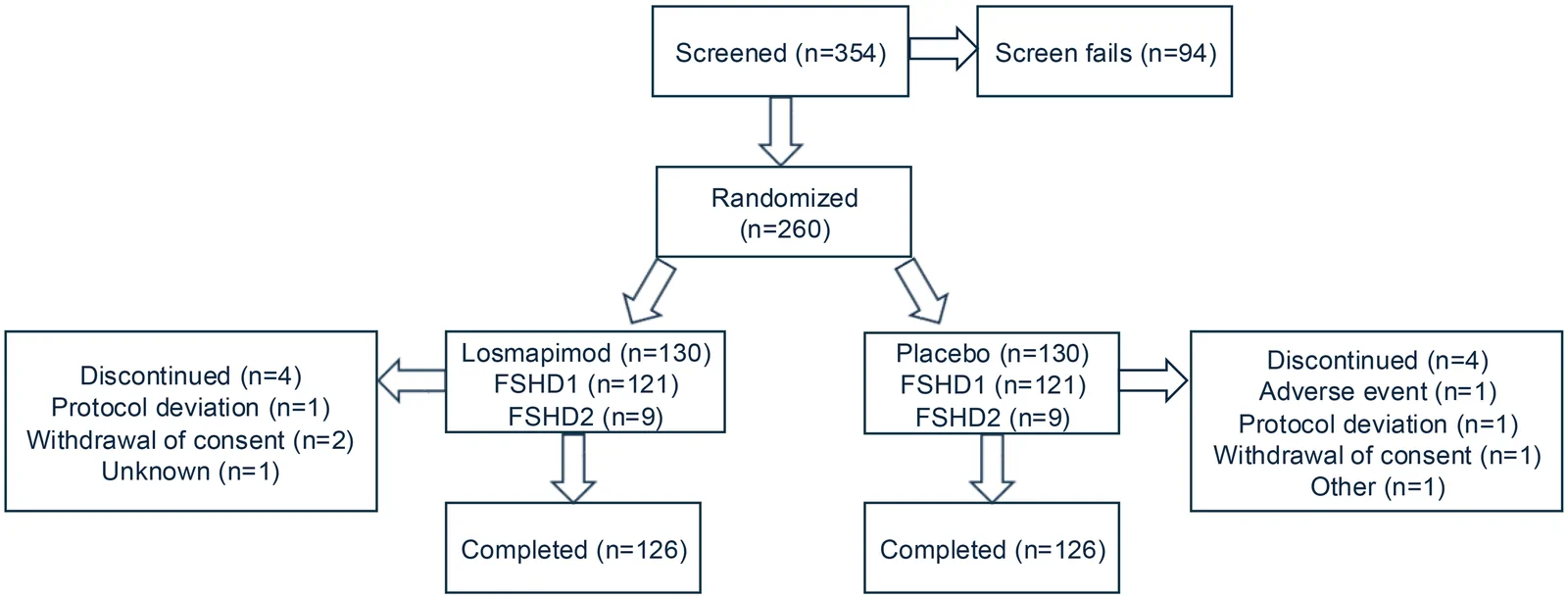
Study design and disposition of participants.

An Independent Data Monitoring Committee was responsible for periodically reviewing data, ensuring participant safety, maintaining overall integrity of the study, and ensuring that any safety issues or recommendations were expeditiously reported to the sponsor Figure 1.

### Study assessments

#### Efficacy

The primary efficacy endpoint was the change from baseline in average total RSA in Q1-Q5 with a 500 g wrist weight at Week 48, where the average is applied over both upper extremities (Q1-Q4 are the four anterior-facing quadrants; Q5 is a single rear- and inferior-facing quadrant). RSA is the outcome measure of the RWS assessment. During the RWS assessment, participants are seated in front of a 3D sensor-based system that tracks each arm's range of motion relative to the torso. The arm's motion in space is visualized by fitting the data onto a spherical model originating at the shoulder joint. RSA describes the portion of the sphere, divided into quadrants (Q1-Q5), covered by each arm's motion.^23^ RSA is a unitless measure, as the surface area covered by the participant's arm is normalized to the participant's arm length, and ranges from 0–1.25 (0–0.25 per quadrant).

Other efficacy and quality of life assessments included whole-body muscle MRI, hand-held dynamometry, PGIC, Patient Global Impression of Severity (PGIS), Quality of Life in Neurologic Disorders – Upper Extremity (Neuro-QoL-UE), FSHD Patient-Reported Outcome (PRO), and Numeric Pain Rating Scale (NPRS). Whole-body musculoskeletal MRI results were analyzed by pre-specified muscle category. Muscles with baseline MFI < 0.10 and MFF < 0.50 were considered category A, muscles with baseline MFI ≥ 0.10 and MFF < 0.50 were considered category B, and muscles with baseline MFF ≥ 0.50 were considered category C ().^12^ These ranges were based on previous work indicating that MFF > 50% is associated with loss of muscle function.^31^ Hand-held dynamometry, which assesses maximal isometric contraction, was used to measure shoulder abduction and hand grip strength (expressed in kg). The PGIC measures participants’ rating of overall improvement compared to the start of the study on a scale of 1 (very much improved) to 7 (very much worse). The PGIS measures participants’ rating of disease severity using the following scale: 0 (no symptoms), 1 (mild symptoms), 2 (moderate symptoms), 3 (severe symptoms), 4 (very severe symptoms). The Neuro-QoL-UE measures participants’ rating of quality of life related to upper extremity function based on ability across fine motor and activities of daily living. Participants rate their ability to do each task using the following scale: 1 (unable to do), 2 (able to do with much difficulty), 3 (able to do with some difficulty), 4 (able to do with a little difficulty), and 5 (able to do without any difficulty). The FSHD PRO measures participants’ health-related quality of life specific to FSHD. Participants were asked to rate their ability to perform tasks in a physical function scale and in a symptoms and limitations scale from 1 (unable to do) to 5 (no difficulty). Finally, the NPRS measures participants’ rating of pain intensity in the last week on a scale from 0 (no pain) to 10 (worst possible pain) (timing of assessments summarized in Table 1).

**Table 1.** Schedule of efficacy and quality of life assessments during the study.

Assessment	Screening	Baseline	Week 4	Week 12	Week 24	Week 36	Week 48/ End of Treatment
Reachable workspace	X	X	X	X	X	X	X
Whole-body musculoskeletal MRI	X				X		X
Hand-held dynamometry		X	X	X	X	X	X
PGIC			X	X	X	X	X
PGIS		X	X	X	X	X	X
Neuro-QoL-UE	X	X	X	X	X	X	X
FSHD PRO		X			X		X
NPRS		X	X	X	X	X	X

Abbreviations: FSHD PRO = Facioscapulohumeral Muscular Dystrophy Patient Reported Outcome; MRI = magnetic resonance imaging; Neuro-QoL UE = Quality of Life in Neurologic Disorders, Upper Extremity; NPRS = Numeric Pain Rating Scale; PGIC = Patient Global Impression of Change; PGIS = Patient Global Impression of Severity.

#### Safety

Safety assessments included adverse events, adverse events of special interest, and serious adverse events (SAEs), which were monitored from informed consent through 30 days after the last dose of study drug; clinical laboratory evaluations; 12-lead electrocardiograms; vital signs; and physical examinations. Adverse events of special interest for this study included liver tests that met the criteria for potential drug-induced liver injury (alanine aminotransferase [ALT] or aspartate aminotransferase [AST] ≥ 3 × upper limit of normal [ULN] with total bilirubin ≥ 2 × ULN, and alkaline phosphatase [ALP] < 2 × ULN).

#### Statistics

The primary population for efficacy analysis consisted of all randomized participants who received at least one dose of study drug. The safety population consisted of all participants who received any study drug. Participants were enrolled proportionally to epidemiologic incidence, with participants with FSHD1 making up approximately 95% of the study population and participants with FSHD2 making up approximately 5% of the study population. A minimum sample size of 210 participants with FSHD1 was needed for at least 95% power using a 2-sided test at a 0.05 significance level to detect a difference of 0.05 between losmapimod and placebo in change from baseline in average total RSA Q1-Q5 with 500 g wrist weight at study week 48, assuming the within-group standard deviation of change was 0.08 and that 10% of the study population would be missing data from study week 48. Screening and randomization procedures at the clinical sites led to over-enrollment with randomization of 260 participants.

The primary endpoint was analyzed using a mixed-effects model for repeated measures (MMRM), with the change from baseline in average total RSA Q1-Q5 with 500 g wrist weight at each post-baseline visit as the dependent variable. The model included terms for treatment group, visit, treatment group by visit interaction, baseline value, baseline value by visit interaction, region, and FSHD repeat number category, where appropriate. An unstructured covariance matrix was used to model the correlations between repeated measurements within each participant. The Kenward-Roger approximation was used to estimate the denominator degrees of freedom. In addition, a linear mixed-effects model (LME) was used to analyze data on average total RSA Q1-Q5 with 500 g wrist weight over the placebo-controlled treatment period to estimate the slope of trend line and % change/year for each treatment group, and differences of estimated slopes and % changes/year between treatment groups.

Other endpoints were summarized using descriptive statistics. Continuous endpoints with multiple post-baseline assessments were analyzed using an MMRM. They were also analyzed using an LME model where appropriate. Relative change from baseline was calculated as 100 × (post-baseline value – baseline value) / baseline value and was analyzed using the Wilcoxon rank-sum test, stratified by FSHD repeat number category. The Hodges-Lehmann estimate of the associated difference and asymptotic nonparametric 95% confidence interval was computed. For all participants, changes in measurements were calculated relative to measurements obtained at baseline. All reported p values (except for those for the primary endpoint) are nominal.

For prespecified subgroup analyses (FSHD1 repeat categories 1–3; FSHD1 repeat categories 4–9; FSHD2; baseline clinical severity score [Ricci score 2–3 and 3.5–4; region [North America and Europe]; sex [male and female]; and age [< 45 years old and ≥ 45 years old], descriptive statistics are reported.

## Results

### Study participants

Of the 354 screened patients, 260 were eligible and randomized to losmapimod treatment (130 participants) or placebo treatment (130 participants). Among the 94 patients who were screened but not randomized the main reasons for screen failure were: screening RSA outside the allowed range (26.6%), baseline RSA outside the allowed range (22.3%), Ricci score outside the allowed range (13.8%), contraindication to MRI (8.5%), history of liver disease or abnormal liver function tests (5.3%), and history of alcohol, analgesic/opioid, and/or illicit drug abuse or positive drug test (5.3%). A total of 252 participants completed the study period and only 4 four participants discontinued in each treatment group. The mean age was 43.9 years and was similar in the losmapimod and placebo groups. In line with epidemiological data, 93.1% of enrolled participants had FSHD1 and 6.9% of enrolled participants had FSHD2. The treatment groups were well balanced in terms of race, ethnicity, FSHD type, and age of disease onset and diagnosis; 37.7% of participants in the losmapimod group and 48.5% in the placebo group had a baseline Ricci severity score of 3.5–4. 15.4% of participants in the losmapimod group and 10.8% in the placebo group had 1–3 D4Z4 repeats. Treatment compliance assessed by tablet counts was >95% in both groups (95.43% and 96.45% in the losmapimod and placebo groups, respectively) (Table 2).

**Table 2.** Demographics and disease baseline characteristics of participants.

	Losmapimod 15 mg BID (n = 130)	Placebo BID (n = 130)	Total (n = 260)
**Age (years)**			
N	130	130	260
Mean (SD)	43.4 (12.3)	44.3 (12.0)	43.9 (12.2)
Median	44.0	44.5	44.0
Min, Max	18, 65	19, 65	18, 65
**Sex (n, %)**			
Male	74 (56.9)	71 (54.6)	145 (55.8)
Female	56 (43.1)	59 (45.4)	115 (44.2)
**Race (n, %)**			
White / Caucasian	115 (88.5)	116 (89.2)	231 (88.8)
Black / African American	3 (2.3)	0	3 (1.2)
Asian	3 (2.3)	3 (2.3)	6 (2.3)
Other	5 (3.8)	4 (3.1)	9 (3.5)
Not reported	4 (3.1)	7 (5.4)	11 (4.2)
**Ethnicity (n, %)**			
Hispanic or Latino	8 (6.2)	6 (4.6)	14 (5.4)
Not Hispanic or Latino	113 (86.9)	112 (86.2)	225 (86.5)
Other	2 (1.5)	5 (3.8)	7 (2.7)
Not reported	7 (5.4)	7 (5.4)	14 (5.4)
**Region n (%)**			
Europe	72 (55.4)	83 (63.8)	155 (59.6)
North America	58 (44.6)	47 (36.2)	105 (40.4)
**FSHD type n (%)**			
FSHD1	121 (93.1)	121 (93.1)	242 (93.1)
1–3 repeats	20 (16.5)	14 (11.6)	34 (14.0)
4–9 repeats	101 (83.5)	107 (88.4)	208 (86.0)
FSHD2	9 (6.9)	9 (6.9)	18 (6.9)
**Ricci score category n (%)**			
2–3	81 (62.3)	67 (51.5)	148 (56.9)
3.5–4	49 (37.7)	63 (48.5)	112 (43.1)
**Age at first symptom (years)**			
N	130	130	260
Mean (SD)	19.9 (11.3)	20.3 (11.7)	20.1 (11.5)
Median	17.0	16.5	17.0
Min, Max	1, 57	0, 62	0, 62

Abbreviations: BID = twice daily; FSHD = facioscapulohumeral muscular dystrophy; SD = standard deviation.

Note: The denominator for all percentages is the number of participants in each treatment group, except for 1–3 repeats and 4–9 repeats whose denominator is the number of participants with FSHD1 in each treatment group.

### Primary efficacy outcome: reachable workspace

**Figure 2.**
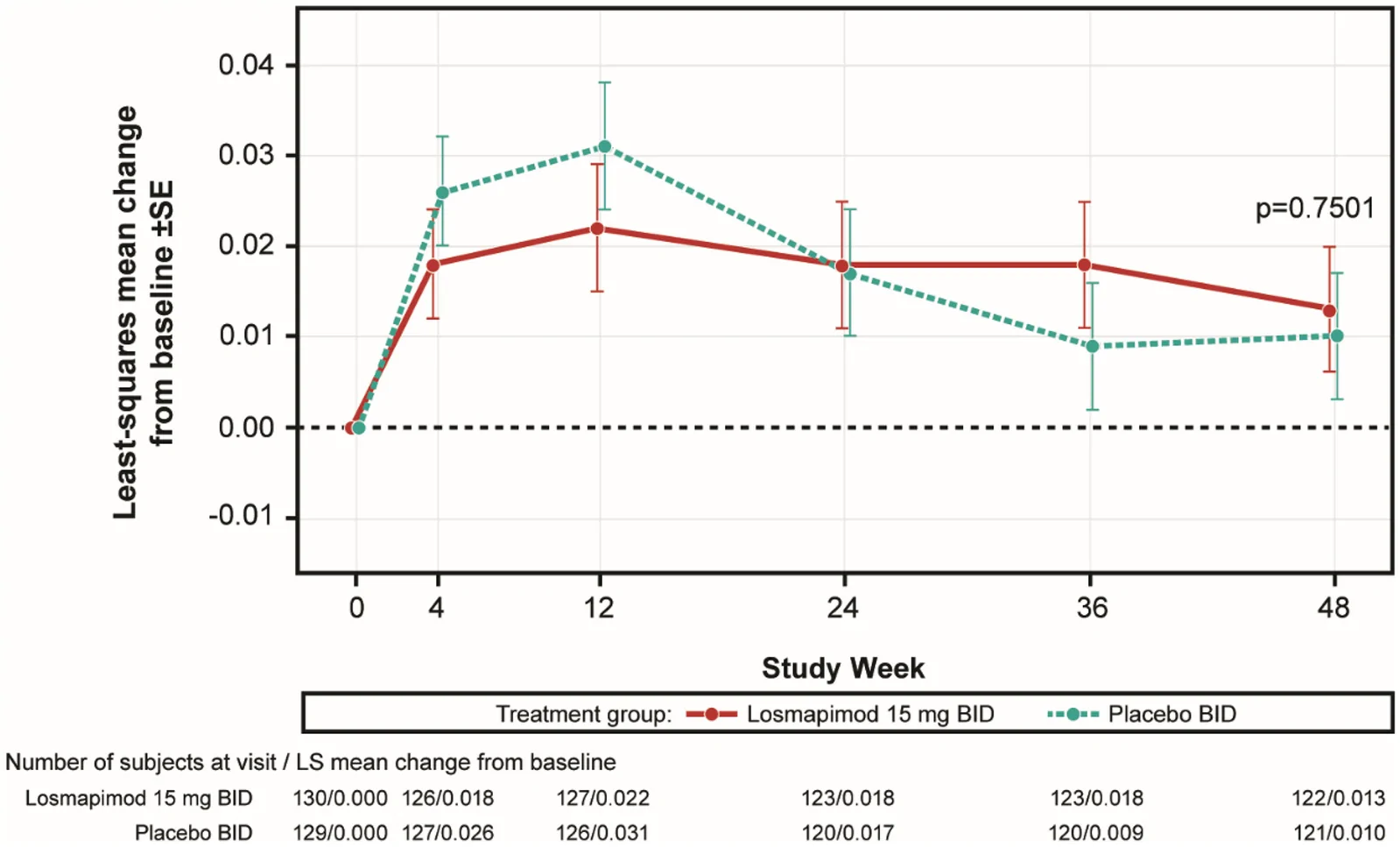
Change from baseline in average total RSA, Q1-Q5 with 500 g wrist weight, based on the reachable workspace assessment.

Abbreviations: BID = twice daily; LS = least-squares; RSA = relative surface area; SE = standard error.

RSA scores were stable or increased in both groups over time (Figure 2 and Suppl Table X). The mean (standard deviation [SD]) total RSA Q1-Q5 with 500 g wrist weight scores at baseline were 0.508 (0.170) and 0.531 (0.161) in the losmapimod and placebo groups, respectively. The mean (SD) change from baseline at Week 48 was 0.011 (0.070) in the losmapimod group and 0.005 (0.068) in the placebo group; the difference in least squares means (2-sided 95% confidence interval [CI]) was 0.003 (−0.014, 0.020), p = 0.7501 (Supplemental Table 2). Therefore, the primary endpoint for this study was not met. Subsequently reported p values are nominal.

### Whole-Body musculoskeletal MRI

**Figure 3.**
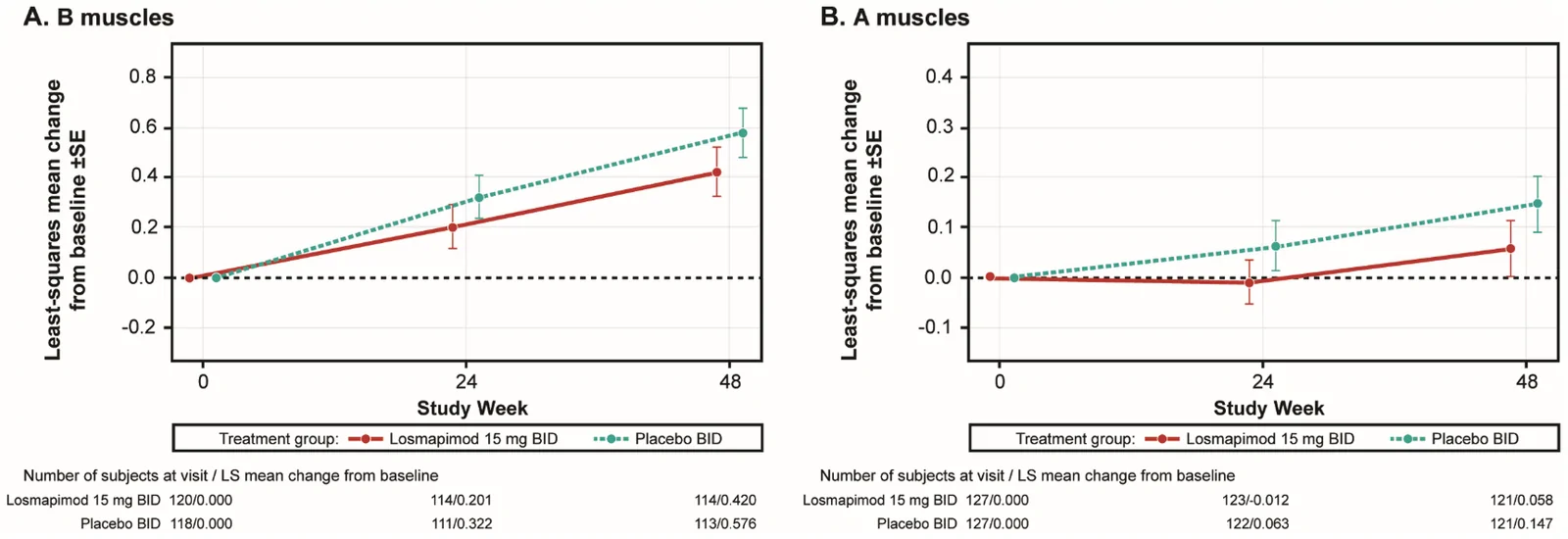
Change from baseline in whole-body longitudinal MFI (%) through week 48 in B muscles **(A)** and in A muscles **(B).**

Abbreviations: BID = twice daily; LS = least-squares; MFI = muscle fat infiltration; SE = standard error.

Baseline MFI in B muscles was similar between treatment groups (mean [SD] losmapimod group: 13.9 [2.1]; placebo: 14.2 [2.1]) (Table 3, Figure 3(A)). MFI increased in both groups over time with a numerically smaller increase in the losmapimod group than the placebo group; the mean (SD) change from baseline in MFI at Week 48 was 0.4 (0.9) in the losmapimod group and 0.6 (0.771) in the placebo group. The difference in least squares means (2-sided 95% CI) was −0.2 (–0.4, 0.1), p = 0.1580. Similar trends were observed for MFI in A muscles (Table 3, Figure 3(B)).

**Table 3.** Whole-body musculoskeletal MRI parameters. Baseline indicates the last non-missing evaluation prior to the first dose of study drug and Week 48 indicates the last visit during the treatment period.

	Losmapimod	Placebo
**MFI (%)**		
**A muscles**		
Baseline, n	127	127
Baseline, mean (SD)	6.2 (1.2)	6.2 (1.1)
Week 48, n	121	121
Change from baseline at Week 48, mean (SD)	0.1 (0.5)	0.2 (0.5)
Difference in LS means (2-sided 95% CI) 2-sided p-value vs placebo	−0.09 (−0.2, 0.03) 0.1513	
**B muscles**		
Baseline, n	120	118
Baseline, mean (SD)	13.9 (2.1)	14.2 (2.1)
Week 48, n	114	113
Change from baseline at Week 48, mean (SD)	0.4 (0.9)	0.6 (0.8)
Difference in LS means (2-sided 95% CI) 2-sided p-value vs placebo	−0.2 (−0.4, 0.1) 0.1580	
**MFF (%)**		
**A muscles**		
Baseline, n	127	127
Baseline, mean (SD)	10.8 (3.6)	10.8(3.7)
Week 48, n	121	121
Change from baseline at Week 48, mean (SD)	0.6 (1.0)	0.9 (1.2)
Difference in LS means (2-sided 95% CI) 2-sided p-value vs placebo	−0.3 (−0.5, −0.004) **0.0470**	
**B muscles**		
Baseline, n	120	118
Baseline, mean (SD)	30.1 (6.9)	31.8 (6.1)
Week 48, n	114	113
Change from baseline at Week 48, mean (SD)	1.9 (2.3)	2.4 (2.2)
Difference in LS means (2-sided 95% CI) 2-sided p-value vs placebo	−0.4 (−1.0, 0.2) 0.1462	
**LMV (L)**		
**A muscles**		
Baseline, n	127	127
Baseline, mean (SD)	5.1 (3.9)	4.6 (3.6)
Week 48, n	121	121
Relative change from baseline at Week 48, mean (SD)	−0.7 (3.5)	−1.5 (3.3)
Difference in LS means (2-sided 95% CI) 2-sided p-value vs placebo	0.8 (−0.09, 1.7) 0.0772	
**B muscles**		
Baseline, n	120	118
Baseline, mean (SD)	1.7 (1.1)	1.9 (1.2)
Week 48, n	114	113
Relative change from baseline at Week 48, mean (SD)	−3.1 (5.8)	−4.4 (4.7)
Difference in LS means (2-sided 95% CI) 2-sided p-value vs placebo	1.5 (0.2, 2.8) **0.0288**	

Abbreviations: CI = confidence interval; LMV = lean muscle volume; LS = least squares; MFF = muscle fat fraction; MFI = muscle fat infiltration; MRI = magnetic resonance imaging; SD = standard deviation.

Note: Bolded p values are nominally significant.

**Figure 4.**
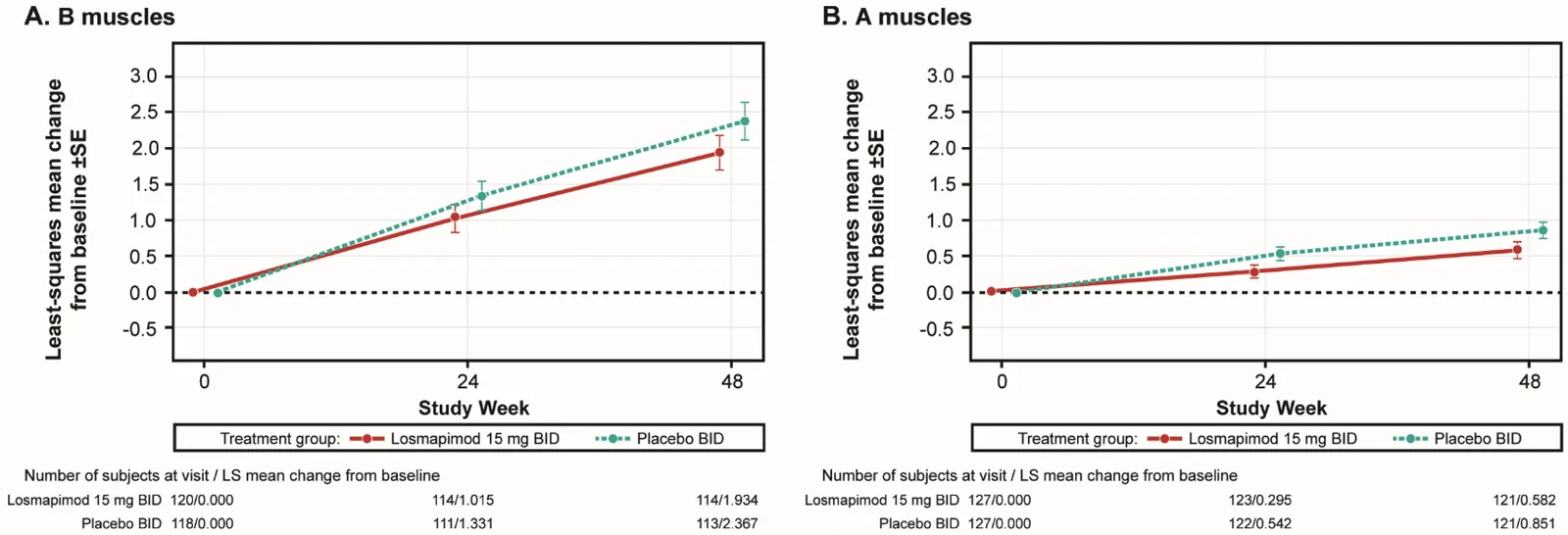
Change from baseline in whole-body longitudinal MFF (%) through week 48 in B muscles **(A)** and in A muscles **(B).**

Abbreviations: BID = twice daily; LS = least-squares; MFF = muscle fat fraction; SE = standard error.

Baseline MFF in B muscles was similar between treatment groups (mean [SD] losmapimod group: 30.1 [6.9]; placebo: 31.8 [6.1]) (Table 3, Figure 4(A)). MFF increased in both groups over time with a numerically smaller increase in the losmapimod group than the placebo group; the mean (SD) change from baseline in MFF at Week 48 was 1.9 (2.3) in the losmapimod group and 2.4 (2.2) in the placebo group. The difference in least squares means (2-sided 95% CI) was −0.4 (−1.0, 0.2), p = 0.1462. Similar trends were observed for MFF in A muscles (Table 3, Figure 4(B)).

**Figure 5.**
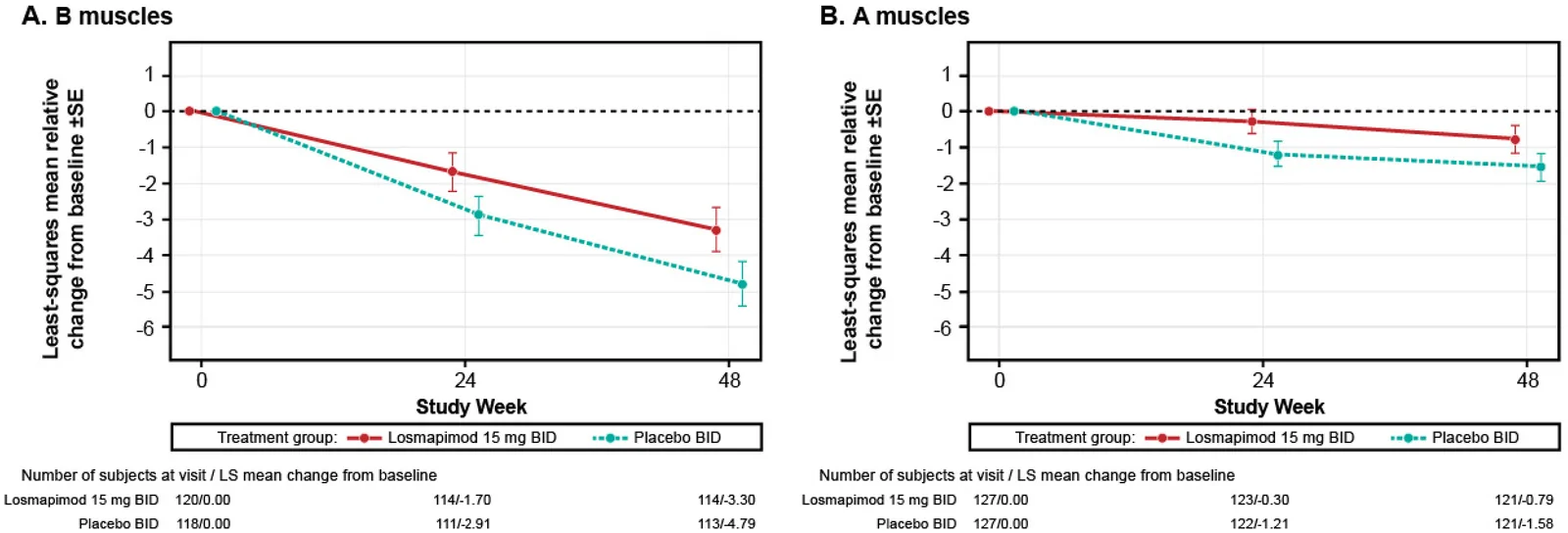
Relative change from baseline in whole-body longitudinal LMV (%) through week 48 in B muscles **(A)** and in A muscles **(B).**

Abbreviations: BID = twice daily; LMV = lean muscle volume; LS = least-squares; SE = standard error.

Baseline LMV (L) in B muscles was similar between treatment groups (mean [SD] losmapimod group: 1.7 [1.1]; placebo: 1.9 [1.2]) (Table 3, Figure 5(A)). LMV decreased in both groups over time with a numerically smaller decrease in the losmapimod group than the placebo group; the mean (SD) relative change from baseline in LMV at Week 48 was −3.1% (5.8%) in the losmapimod group and −4.4% (4.7%) in the placebo group. The difference in least squares means (2-sided 95% CI) was 1.5% (0.2%, 2.8%), p = 0.0288. Similar trends were observed for LMV in A muscles (Table 3, Figure 5(B)).

### Muscle strength

Average shoulder abduction strength as assessed by hand-held quantitative dynamometry increased in both groups over time; the mean (SD) relative change from baseline at Week 48 was 17.38% (52.17%) in the losmapimod group and 16.87% (56.81%) in the placebo group. The H-L estimate of treatment difference (2-sided 95% CI) was 3.84% (–5.82%, 14.41%), p = 0.5067 (Table 4).

**Table 4.** Muscle strength (kg) as assessed by quantitative dynamometry.

	Losmapimod	Placebo
	N = 130	N = 130
**Average shoulder abductor strength (kg)**		
Baseline, n	129	129
Baseline, mean (SD)	5.05 (2.95)	5.32 (3.99)
Week 48, n	124	125
Relative change from baseline at Week 48, mean (SD) (%)	17.38 (52.17)	16.87 (56.81)
H-L estimate of treatment difference (2-sided 95% CI) 2-sided p-value vs placebo	3.84 (−5.82, 14.41) p = 0.5067	
**Average hand grip strength (kg)**		
Baseline, n	129	129
Baseline, mean (SD)	25.82 (10.08)	27.07 (10.12)
Week 48, n	124	124
Relative change from baseline at Week 48, mean (SD) (%)	6.32 (26.15)	2.73 (21.34)
H-L estimate of treatment difference (2-sided 95% CI) 2-sided p-value vs placebo	2.19 (−1.35, 6.17) 0.2705	

Abbreviations: CI = confidence interval; H-L = Hodges-Lehmann; SD = standard deviation.

Average hand grip strength as assessed by hand-held quantitative dynamometry also increased in both groups over time with a numerically larger increase in the losmapimod group than the placebo group; the mean (SD) relative change from baseline at Week 48 was 6.32% (26.15%) in the losmapimod group and 2.73% (21.34%) in the placebo group. The H-L estimate of treatment difference (2-sided 95% CI) was 2.19% (−1.35%, 6.17%), p = 0.2705 (Table 4).

### Patient-Reported outcomes

PGIC, PGIS, Neuro-QoL UE, and FSHD PRO Physical Function scores at Week 48 were similar between treatment groups (Table 5). A PGIC score of 4 indicates no change. Losmapimod-treated participants had decreased FSHD PRO Symptoms and Limitations scores (i.e., fewer symptoms and limitations) and NPRS scores (i.e., improvement) compared to placebo-treated participants.

**Table 5.** Scores over time in assessments of patient-reported outcomes.

	Losmapimod	Placebo
	N = 130	N = 130
**Patient Global Impression of Change (scale: 1–7)**		
Week 48, n	125	126
Week 48, mean (SD)	4.1 (1.1)	4.2 (1.1)
Difference in LS means (2-sided 95% CI) 2- sided p-value vs placebo	−0.1 (–0.4, 0.2) p = 0.5082	
**Patient Global Impression of Severity (scale: 0–4)**		
Baseline, n	120	125
Baseline, mean (SD)	2.2 (0.7)	2.1 (0.8)
Week 48, n	116	122
Change from baseline at Week 48, mean (SD) (%)	−0.1 (0.8)	0.0 (0.8)
Difference in LS means (2-sided 95% CI) 2- sided p-value vs placebo	−0.2 (–0.3, 0.0) 0.0775	
**Quality of Life in Neurologic Disorders, Upper Extremity (scale: 1–5 on each of the 20 items)**		
Baseline, n	126	130
Baseline, mean (SD)	74.12 (5.60)	74.34 (5.88)
Week 48, n	120	125
Change from baseline at Week 48, mean (SD)	−2.10 (3.82)	−1.51 (3.68)
Difference in LS means (2-sided 95% CI) 2- sided p-value vs placebo	−0.54 (–1.49, 0.41) p = 0.2653	
**FSHD PRO Physical Function Scale (scale: 1–5 on each of the 17 items)**		
Baseline, n	122	129
Baseline, mean (SD)	58.52 (11.79)	58.08 (11.62)
Week 48, n	117	124
Change from baseline at Week 48, mean (SD)	−2.13 (5.90)	−1.82 (5.34)
Difference in LS means (2-sided 95% CI) 2- sided p-value vs placebo	−0.32 (–1.74, 1.09) 0.6513	
**FSHD PRO Symptoms and Limitations Scale (scale: 1–5 on each of the 11 items)**		
Baseline, n	122	130
Baseline, mean (SD)	24.0 (7.4)	23.6 (7.2)
Week 48, n	117	125
Change from baseline at Week 48, mean (SD)	−0.7 (5.5)	0.4 (5.3)
Difference in LS means (2-sided 95% CI) 2- sided p-value vs placebo	−1.0 (–2.3, 0.3) 0.1318	
**Numeric Pain Rating Scale (scale: 0–10)**		
Baseline, n	121	128
Baseline, mean (SD)	3.4 (2.4)	3.2 (2.4)
Week 48, n	115	121
Change from baseline at Week 48, mean (SD)	−0.3 (1.9)	0.3 (1.7)
Difference in LS means (2-sided 95% CI) 2- sided p-value vs placebo	−0.5 (–1.0, –0.1) **0.0190**	

Abbreviations: CI = confidence interval; FSHD PRO = Facioscapulohumeral Muscular Dystrophy Patient Reported Outcome; LS = least squares; N = number of participants; SD = standard deviation.

Note: Bolded p values are nominally significant.

### Subgroup analyses

Subgroup analyses were conducted for primary (RWS) and secondary (PGIC, MFI of B muscles, shoulder abductor strength, and Neuro-QoL UE) endpoints. Distribution of participants across subgroups are presented in Table 2. Descriptive statistics for disease subgroup analyses are presented in Table 6.

**Table 6.** Results of secondary endpoints by disease subgroups.

	FSHD1, 1–3 repeats		FSHD1, 4–9 repeats		FSHD2	
	Losmapimod N = 20	Placebo N = 14	Losmapimod N = 101	Placebo N = 107	Losmapimod N = 9	Placebo N = 9
**Average total RSA**						
Baseline, n	20	14	101	106	9	9
Baseline, mean (SD)	0.500 (0.141)	0.524 (0.110)	0.507 (0.180)	0.531 (0.165)	0.543 (0.122)	0.538 (0.185)
Week 48, n	18	13	96	100	8	8
Change from baseline at Week 48, mean (SD)	0.006 (0.064)	0.021 (0.061)	0.010 (0.072)	0.002 (0.070)	0.039 (0.060)	0.015 (0.044)
Difference in LS means (2-sided 95% CI) 2-sided p-value vs placebo	–0.016 (–0.061, 0.030) p = 0.4912		0.003 (–0.017, 0.023) p = 0.7406		0.023 (–0.028, 0.075) p = 0.3498	
**MFI (%) of B muscles**						
Baseline, n	18	13	94	97	8	8
Baseline, mean (SD)	13.915 (2.315)	13.333 (1.596)	13.935 (2.120)	14.266 (2.132)	13.478 (1.873)	15.361 (1.493)
Week 48, n	16	12	91	94	7	7
Change from baseline at Week 48, mean (SD)	0.169 (0.767)	0.925 (1.342)	0.479 (0.898)	0.457 (0.647)	–0.004 (1.006)	1.169 (0.666)
Difference in LS means (2-sided 95% CI) 2-sided p-value vs placebo	–0.810 (–1.604, -0.017) **p** **=** **0.0458**		0.015 (–0.209, 0.239) p = 0.8947		–1.073 (–2.376, 0.230) p = 0.0971	
**Average shoulder abductor strength (kg)**						
Baseline, n	20	14	100	106	9	9
Baseline, mean (SD)	5.24 (2.97)	5.77 (8.62)	4.88 (2.97)	5.30 (3.15)	6.46 (2.45)	4.83 (1.89)
Week 48, n	18	13	98	104	8	8
Relative change from baseline at Week 48, mean (SD)	24.01 (40.38)	16.86 (49.42)	17.45 (55.89)	16.62 (59.23)	1.61 (13.28)	20.09 (36.70)
H-L estimate of treatment difference (2-sided 95% CI) 2-sided p-value vs placebo	7.91 (–14.52, 39.65) p = 0.5616		4.51 (–7.55, 16.32) p = 0.4737		–10.87 (–36.44, 7.31) p = 0.3720	
**Patient Global Impression of Change (scale: 1–7)**						
Week 48, n	18	13	99	105	8	8
Week 48, mean (SD)	3.9 (1.1)	3.8 (1.1)	4.1 (1.1)	4.2 (1.1)	4.0 (1.1)	4.6 (1.2)
Difference in LS means (2-sided 95% CI) 2-sided p-value vs placebo	0.1 (–0.7, 0.9) p = 0.7656		–0.1 (–0.4, 0.2) p = 0.6718		–0.6 (–1.8, 0.6) p = 0.2717	
**Quality of Life in Neurologic Disorders, Upper Extremity (scale: 1–5)**						
Baseline, n	19	14	98	107	9	9
Baseline, mean (SD)	71.37 (5.97)	73.50 (5.71)	74.53 (5.59)	74.34 (6.08)	75.44 (2.96)	75.67 (3.54)
Week 48, n	17	13	95	104	8	8
Change from baseline at Week 48, mean (SD)	–1.12 (5.60)	–1.23 (1.64)	–2.26 (3.36)	–1.47 (3.92)	–2.25 (4.71)	–2.50 (2.67)
Difference in LS means (2-sided 95% CI) 2-sided p-value vs placebo	–0.32 (–3.67, 3.04) p = 0.8487		–0.69 (–1.72, 0.33) p = 0.1832		0.54 (–3.53, 4.60) p = 0.7803	

Abbreviations: CI = confidence interval; FSHD = facioscapulohumeral muscular dystrophy; H-L = Hodges-Lehmann; LS = least squares; MFI = muscle fat infiltration; N = number of participants; RSA = relative surface area; SD = standard deviation.

Note: Bolded p values are nominally significant.

There was no statistical difference at Week 48 between treatment groups in RWS despite numeric imbalance between groups for D4Z4 repeat category, Ricci score category, and geographic distribution category. There were differences at Week 48 between treatment groups in MFI of B muscles analyzed in the 1–3 repeat subgroup with a smaller increase in MFI in the losmapimod group in participants with 1–3 repeats but not in participants with 4–9 repeats (difference in least squares [LS] means [2-sided 95% CI] −0.810 [-1.604, −0.017], p = 0.0458); in the Ricci score 3.5–4 subgroup (difference in LS means [2-sided 95% CI] −0.326 [–0.599, −0.052], p = 0.0203); in the Europe subgroup (difference in LS means [2-sided 95% CI] −0.268 [–0.494, −0.043], p = 0.0200); and in the female subgroup (difference in LS means [2-sided 95% CI] −0.423 [–0.716, −0.130], p = 0.0050). These differences were nominally significant.

There were no differences in subgroup analyses by baseline clinical severity score (Ricci score 2–3 and 3.5–4); region (North America and Europe); sex (male and female); and age (< 45 years old and ≥ 45 years old) at Week 48 between treatment groups for RWS, PGIC, shoulder abduction strength, or Neuro-QoL UE assessments.

### Safety

Safety was similar between both treatment groups. In the losmapimod group, 122 (93.8%) participants experienced at least one treatment-emergent adverse event (TEAE), 5 (3.8%) participants experienced at least one SAE, 37 (28.5%) participants experienced at least one TEAE that was considered related to study drug (possibly, probably, or definitely related), and 1 (0.8%) participant experienced an adverse event (dizziness) leading to treatment discontinuation. In the placebo group, 112 (86.2%) participants experienced at least one TEAE, 8 (6.2%) participants experienced at least one SAE, 32 (24.6%) participants experienced at least one TEAE that was considered related to study drug (possibly, probably, or definitely related), including 1 (0.8%) participant who experienced an SAE that was considered possibly related to study drug, and 1 (0.8%) participant who experienced an adverse event (blood creatinine increase) leading to treatment discontinuation. There were no adverse trends in serum chemistry, hematology, urinalysis, or QTcF intervals throughout the study. There were no drug-induced liver injuries reported.

One participant (0.8%) in the placebo group died due to metastatic small cell lung cancer; this participant had experienced the SAE of serum creatinine increase. This death occurred during the treatment period and was deemed unrelated to study drug.

The most frequently reported TEAEs considered related to study drug (possibly, probably, or definitely related) were headache (12 participants; 4.6%), diarrhea (7 participants; 2.7%), nausea (5 participants; 1.9%), dyspepsia and fatigue (4 participants each; 1.5%), rash and dry skin (3 participants each; 1.2%). The following SAEs were reported: appendicitis (2 participants; 1.5%) and COVID-19, pneumonia, urosepsis, accidental exposure to product by child of participant, ankle fracture, meniscus injury, pain in extremity, spinal stenosis, extrasystoles, ischemic colitis, blood creatinine increased, metastatic small cell lung cancer, and lumbar radiculopathy (1 participant each; 0.8%). Only the SAE of accidental exposure to product by child was considered possibly related to study drug and occurred in the placebo group. The TEAEs leading to study drug discontinuation were dizziness (1 participant [0.8%] in the losmapimod group) and blood creatinine increased (1 participant [0.8%] in the placebo group) Table 7.

**Table 7.** Overall safety and tolerability of losmapimod.

	Losmapimod N (%) participants	Placebo N (%) participants
**Overall summary, n (%)**		
At least one TEAE	122 (93.8)	112 (86.2)
At least one SAE	5 (3.8)	8 (6.2)
TEAEs related to study drug	37 (28.5)	32 (24.6)
SAEs related to study drug	0	1 (0.8)
TEAEs of CTCAE Grade ≥ 3	8 (6.2)	12 (9.2)
TEAEs leading to treatment discontinuation	1 (0.8)	1 (0.8)
TEAEs leading to death	0	1 (0.8)
Any TEAE of special interest	0	0
**All TEAEs occurring in** **≥** **5% of participants, n (%) [events in analysis]**		
Nasopharyngitis	30 (23.1) [40]	29 (22.3) [40]
Fall	22 (16.9) [55]	23 (17.7) [111]
Headache	16 (12.3) [24]	16 (12.3) [26]
COVID-19	16 (12.3) [17]	13 (10.0) [13]
Diarrhea	9 (6.9) [11]	11 (8.5) [16]
Influenza-like illness	8 (6.2) [8]	1 (0.8) [1]
Nausea	7 (5.4) [7]	7 (5.4) [9]
Dizziness	7 (5.4) [7]	2 (1.5) [4]
Rash	7 (5.4) [7]	1 (0.8) [1]
Fatigue	7 (5.4) [7]	7 (5.4) [7]
Influenza	6 (4.6) [7]	8 (6.2) [8]
Arthralgia	6 (4.6) [6]	12 (9.2) [15]
Pain in extremity	6 (4.6) [6]	11 (8.5) [14]

Abbreviations: CTCAE = Common Terminology Criteria for Adverse Events; SAE = serious adverse event; TEAE = treatment-emergent adverse event.

## Discussion

There are currently no approved therapies targeting the pathophysiology of FSHD. Losmapimod was hypothesized to slow disease progression and/or improve muscle function by reducing *DUX4* expression in skeletal muscle of both FSHD1 and FSHD2. A previous phase 2 clinical study with losmapimod, ReDUX4, was not able to demonstrate changes in DUX4-driven gene expression in muscle biopsies of losmapimod-treated participants compared to placebo-treated participants.^20^ However, improvements in functional outcomes and fatty muscle replacement (RWS, PGIC, musculoskeletal MRI) were observed in the losmapimod group, which motivated the present phase 3 study. Analysis of the gene expression data showed high variability of gene expression profiles, both across- and within-subjects. This variability was greater than was anticipated based on prior exploratory studies. A number of factors are thought to have played a role in this variability: a) the stochastic nature of DUX4 expression in myonuclei, b) the variable and heterogeneous proportion of tissues collected in each muscle biopsy sample (fat, muscle tissue, fibrous tissue, inflammatory cells), c) variability in the biopsy procedure, and d) potential expression changes associated with the biopsy process itself (as opposed to changes associated with the underlying disease +/–drug effect). Improvements in muscle biopsy and gene expression analysis techniques directed towards minimizing variability and increasing reproducibility could make this a more robust approach to de-risk future longer-term clinical studies in FSHD, but as conducted in ReDUX4, the data were not sufficiently compelling to continue using this evaluation in the REACH study.The REACH study would have been the first registrational study in FSHD, meaning that there are no established endpoints to demonstrate efficacy of a therapy in this indication. Based on published evidence and because patients with FSHD characteristically experience shoulder weakness, RWS was considered to be capable of capturing changes in upper extremity function over time.^19,20,26,27^ However, the REACH study was the first registrational study in any indication to use RWS as an assessment for an efficacy endpoint.

Unexpectedly, participants in both the losmapimod and placebo groups demonstrated some improvement in RWS during the study contrasting with the decline observed in the placebo group in ReDUX4. This may reflect a placebo effect to which various factors may have contributed in the REACH study. A previous analysis of phase 3 studies of other therapies for FSHD showed that participants enrolled in clinical studies outperformed participants in natural history studies in measures of strength regardless of treatment assignment (active treatment or placebo), which likely reflects patients’ expectations of treatment benefit.^32^ In progressive disorders with no effective treatments, patients’ high expectations of benefit (therapeutic misestimation) may contribute to a placebo response.^33^ Future studies should focus on preventing therapeutic misestimation by managing patient expectations regarding trial outcomes. This involves early and continuous education on the limitations of clinical trials, emphasizing that not all patients may benefit and that lost function may not be restored. Additionally, using neutral terms such as “study compound” instead of “treatment” and discussing the possibility of neutral outcomes during the consent process are essential strategies. In addition, the improvements in RWS in both losmapimod and placebo groups during the conduct of the study could reflect a learning effect. A longer run-in period could mitigate the learning effect by allowing participants to acclimate to trial assessments such as RWS, reducing the impact of initial familiarity.

Additionally, the 48-week duration of this study, despite being based on ReDUX4 results, may have been too short to demonstrate a treatment benefit when compared to the rate of natural progression of FSHD, suggesting a possible placebo effect. The short duration was chosen to avoid delayed access to treatment if REACH had positive outcomes. Further, participants were likely aware of the results of the ReDUX4 study, which may have further contributed to this effect. In particular, participants’ optimism about the possible efficacy of losmapimod may have led to greater effort in performing functional assessments, which are known to be motivation- and effort-dependent.^33^ Increased sample size also may not have allayed the placebo effect, as larger studies can lead to increased participant expectations and are associated with stronger placebo effects.^34^ MRI imaging data, which should not be sensitive to a placebo effect, showed only nominal improvement in the losmapimod group over placebo.

There were a few differences in eligibility criteria between the ReDUX4 and REACH studies, which may have contributed to the difference in efficacy results. While both studies used the same overall disease severity inclusion criterion (Ricci score of 2–4), there were 3 differences that may have contributed to the different results observed in the ReDUX4 and REACH studies. First, to be eligible for the ReDUX4 study, participants were required to have STIR + lesions in a needle biopsy-accessible muscle, as determined by MRI at screening. Muscle biopsy and STIR analysis, were not included in the REACH study as inclusion criteria. Because STIR + lesions are thought to indicate active disease, the two studies may have enrolled qualitatively different patient populations; participants in the REACH study may have had less active disease than those in ReDUX4 and therefore may have been less responsive to losmapimod-induced reduction of DUX4.^13,31^ Second, the REACH, but not ReDUX4, study included participants with FSHD2. However, few participants with FSHD2 were enrolled in the REACH study (N = 18), these participants were equally distributed across treatment groups. Finally, patients were required to have RSA scores ≥ 0.2 and ≤ 0.7 to be eligible for REACH (this was not included as an eligibility criterion in the ReDUX4 study). This inclusion criterion was selected to enroll moderately impaired participants who were most likely to experience measurable change in RWS over time, thereby avoiding floor and ceiling effects that may have appeared with the enrollment with the most and least severely affected patients. The FSHD patient population is known to be heterogenous in terms of severity and progression of muscle weakness, magnifying differences in eligibility criteria across studies. It is unclear whether, and to what extent, these differences could have contributed to the difference in efficacy observed in the ReDUX4 and REACH studies. Future work is needed to determine the best way of accounting for heterogeneity among patients.

This study emphasized upper extremity muscles and RWS. One assessment of lower limb mobility, the Timed Up and Go (TUG) assessment, showed variable results over time in the ReDUX4 study and was therefore not included in the REACH study. Other, more sensitive functional outcome assessments of lower extremity muscles should be considered in future studies.

## Conclusions

Losmapimod was generally well tolerated with a favorable safety profile at a dose of 15 mg twice daily. Although the REACH study did not meet its primary endpoint, study design and data from the study may inform future studies of FSHD therapies.

## Supplemental Material

sj-docx-1-jnd-10.1177_22143602261419558 - Supplemental material for A randomized, double-blind, placebo-controlled study of losmapimod in patients with facioscapulohumeral muscular dystrophy: Results of the REACH study Supplemental material, sj-docx-1-jnd-10.1177_22143602261419558 for A randomized, double-blind, placebo-controlled study of losmapimod in patients with facioscapulohumeral muscular dystrophy: Results of the REACH study by Nicol C Voermans, Jeffrey M Statland, Lawrence J Hayward, Angela Rosenbohm, Adolfo López de Munain, Sabrina Sacconi, Doris G Leung, Umesh A Badrising, John Vissing, Benedikt Schoser, Nuria Muelas, Hanns Lochmüller, Enrico Bugiardini, Leo H Wang, Lorenzo Maggi, Thomas Ragole, Alan Pestronk, Johanna I Hamel, Namita A Goyal, Lawrence Korngut, Elie Naddaf, Amy Harper, Perry B Shieh, Cornelia Kornblum, Valeria Sansone, Angela Genge, Giorgio Tasca, John Jiang, Marie-Helene Jouvin, Rabi Tawil and in Journal of Neuromuscular Diseases

sj-docx-2-jnd-10.1177_22143602261419558 - Supplemental material for A randomized, double-blind, placebo-controlled study of losmapimod in patients with facioscapulohumeral muscular dystrophy: Results of the REACH study Supplemental material, sj-docx-2-jnd-10.1177_22143602261419558 for A randomized, double-blind, placebo-controlled study of losmapimod in patients with facioscapulohumeral muscular dystrophy: Results of the REACH study by Nicol C Voermans, Jeffrey M Statland, Lawrence J Hayward, Angela Rosenbohm, Adolfo López de Munain, Sabrina Sacconi, Doris G Leung, Umesh A Badrising, John Vissing, Benedikt Schoser, Nuria Muelas, Hanns Lochmüller, Enrico Bugiardini, Leo H Wang, Lorenzo Maggi, Thomas Ragole, Alan Pestronk, Johanna I Hamel, Namita A Goyal, Lawrence Korngut, Elie Naddaf, Amy Harper, Perry B Shieh, Cornelia Kornblum, Valeria Sansone, Angela Genge, Giorgio Tasca, John Jiang, Marie-Helene Jouvin, Rabi Tawil and in Journal of Neuromuscular Diseases
