# Supplementary material for: A randomized, double-blind, placebo-controlled study of losmapimod in patients with facioscapulohumeral muscular dystrophy: Results of the REACH study
Source: J Neuromuscul Dis. 2026 Feb 6;13(5):1049–64. doi: 10.1177/22143602261419558 (PMC13442687; doi:10.1177/22143602261419558)
Supplement: Supplementary file 1 [file sj-docx-1-jnd-10.1177_22143602261419558.docx]

**Supplemental Table 1.** Summary of inclusion and exclusion criteria for Parts A and B.

| Part A: Placebo-Controlled Treatment Period | | **Part B: Open-Label Extension** | |
| --- | --- | --- | --- |
| **Inclusion Criteria** | **Exclusion Criteria** | **Inclusion Criteria** | **Exclusion Criteria** |
| 1. Patient will sign and date an informed consent form (ICF). 2. Patients will have a diagnosis of FSHD1 or FSHD2 verified by genetic testing.    - Randomization will be stratified for FSHD1 to ensure that treatment allocation is balanced across FSHD repeat number categories (i.e., 1 to 3 repeats versus 4 to 9 repeats).    - Randomization will be stratified for FSHD2 to ensure that an equal number of patients will be allocated to treatment and placebo. 3. Patients will have a Clinical Severity Score of 2 to 4 (Ricci score; range 0 to 5) at screening. Patients who are wheelchair-dependent or dependent on walker or wheelchair for activities are not permitted to enroll in the study. 4. Patients with screening total RSA (Q1-Q4) without weight in the dominant UE assessed by RWS ≥ 0.2 and ≤ 0.7. 5. Willing and able to comply with scheduled visits, treatment plan, study restrictions, laboratory tests, contraceptive guidelines, and other study procedures. 6. No contraindications to MRI. 7. Patients (male and female) will be between the ages of 18 and 65 years at the time of consent, inclusive.    - A female patient is eligible to participate if she is of non-childbearing potential, defined as pre-menopausal females with a documented hysterectomy, bilateral salpingectomy, or bilateral oophorectomy; if she is postmenopausal, defined as no menses for 12 months without an alternative medical cause; OR    - If of child-bearing potential, she is using a highly effective method for avoidance of pregnancy for the duration of dosing and until 90 days after the last dose.    - Male patients must agree to use one of a contraception method. This criterion must be followed from the time of the first dose of study medication and until 90 days after the last dose of study drug. | 1. History of any illness or any clinical condition that, in the opinion of the Investigator, might confound the results of the study or pose an additional risk in administering study drug to the patient. This may include, but is not limited to, history of relevant drug or food allergies; history of cardiovascular or central nervous system disease; history or presence of clinically significant pathology; clinically significant history of mental disease. 2. Previously diagnosed cancer that has not been in complete remission for at least 5 years. Localized carcinomas of the skin and carcinoma *in situ* of the cervix that have been resected or ablated for cure are not exclusionary. 3. For patients who are on drug(s) or supplements that may affect muscle function, as determined by the Investigator, or are predetermined in the study design: patients must be on a stable dose of that drug(s) or supplement for at least 3 months prior to the first dose of study drug and remain on that stable dose for the duration of the study. Changes to the dose or treatment discontinuation during the study can only be done for strict medical reasons by the treating physician with clear documentation and notification to the Sponsor. 4. History of febrile illness within 5 days before the first study drug dose. Patients who were healthy during screening but develop febrile illness in the 5 days before randomization need to have the baseline visit postponed until the febrile illness is fully resolved (fever-free without the use of antipyretic agents for 24 hours). Once the febrile illness is fully resolved, the patient’s baseline visit can be scheduled. The duration of the screening period in such cases can be extended to up to 35 days. 5. Known active opportunistic or life-threatening infections including HIV and hepatitis B or C. 6. Known active or inactive tuberculosis infection. 7. Current acute liver disease or chronic liver disease as defined by any of the following:  - Current ALT ≥ 2 × upper limit of normal (ULN) or total bilirubin > 1.5 × ULN (unless participant has Gilbert’s syndrome characterized by the combination of total bilirubin < 3 × ULN, direct bilirubin within the normal range and normal ALT and AST, or the presence of mutations in the UDP-glucuronosyltransferase 1 gene, indicative of Gilbert’s syndrome). - Positive for hepatitis B surface antigen. - Positive for hepatitis C antibody unless additional testing for hepatitis C viral RNA is negative, ALT is < 2 × ULN and total bilirubin is ≤ 1.5 × ULN, indicating inactive/resolved hepatitis C infection.  8. Known severe renal impairment (defined as a glomerular filtration rate of < 30 mL/min/1.73 m^2^). 9. Standard 12-lead ECG demonstrating QTcF >450 msec for male patients and QTcF >470 msec for female patients at screening. If QTcF exceeds 450 msec for males or 470 msec for females, the ECG will be repeated 2 more times, and the average of the 3 QTcF values will be used to determine the patient’s eligibility. 10. History of cardiac dysrhythmias requiring anti-arrhythmia treatment(s); or history or evidence of abnormal ECGs that, in the opinion of the Investigator or Medical Monitor, would preclude the patient’s participation in the study. 11. Male patients with a female partner who is planning to become pregnant during the study or within 90 days after the last study drug dose. 12. Concomitant use of cytotoxic chemotherapy for cancer or known ongoing or anticipated use of chronic severe immunosuppressive agents. 13. Positive pregnancy test, known to be pregnant or lactating, or planning to become pregnant during study drug administration and until 90 days after last dose. 14. Any current mental condition (psychiatric disorder, senility or dementia) that may affect study compliance or prevent understanding of the aims, investigational procedures, or possible consequences of the study. 15. Patient has any condition possibly affecting drug absorption (e.g., gastrectomy, cholecystectomy, or other gastrointestinal tract surgery, except appendectomy). 16. History of alcohol, analgesic/opioid, and/or illicit drug abuse, as defined by the Diagnostic and Statistical Manual of Mental Disorders, Fifth Edition (American Psychiatric Association, 2013), in the last 6 months before screening or a positive test for drugs of abuse at screening. Use of CBD/THC is permitted. 17. Use of another investigational product within 30 days or 5 half-lives (whichever is longer) or according to local regulations or currently participating in a study of an investigational device. 18. Current or anticipated participation in a natural history study. Previous participation is allowed but patients cannot continue after enrollment in Study 1821-FSH-301. 19. Known hypersensitivity or intolerance to losmapimod or any of its excipients. 20. Previous participation in a Fulcrum-sponsored FSHD losmapimod study (FIS-001-2019 or FIS-002-2019). 21. Anticipated inability to comply with any study procedures, including participation in study visits according to the visit schedule through 48 weeks. 22. Abnormal laboratory results indicative of any significant medical disease that, in the opinion of the Investigator, would preclude the patient's participation in the study. 23. Patient, or close relative of the patient, is the Investigator or a subinvestigator, research assistant, pharmacist, study coordinator, or other staff directly involved with the conduct of the study at that site. 24. Patient is vulnerable (i.e., deprived of freedom), including inmates of psychiatric wards and prison or state institutions, patients with commitments to an institution, or a patient who is detained or committed to an institution by a law court or by legal authorities. 25. ***For Italy only:*** Vaccination with a live attenuated vaccine within 6 weeks prior to randomization until the safety follow-up visit | 1. Patient completed 48 weeks of treatment during Part A. 2. Patient will sign and date an ICF. 3. Patient is willing and able to comply with scheduled visits, treatment plan, study restrictions, laboratory tests, contraceptive guidelines, and other study procedures. 4. Patient agrees to the following methods of contraception:    - Female patients of childbearing potential agree to continue using a highly effective method for avoidance of pregnancy for the duration of dosing and until 90 days after the last dose.    - Male patients must agree to use a contraception method. This criterion must be followed from the time of the first dose of study medication and until 90 days after the last dose of study drug. | 5. Any clinical condition that, in the opinion of the Investigator, might confound the results of the study or pose an additional risk in administering study drug to the patient. 6. Male patients with a female partner who is planning to become pregnant during the study or within 90 days after the last study drug dose. 7. Anticipated inability to comply with any study procedures, including participation in study visits. |
