# Supplementary material for: A randomized, double-blind, placebo-controlled study of losmapimod in patients with facioscapulohumeral muscular dystrophy: Results of the REACH study
Source: J Neuromuscul Dis. 2026 Feb 6;13(5):1049–64. doi: 10.1177/22143602261419558 (PMC13442687; doi:10.1177/22143602261419558)
Supplement: Supplementary file 2 [file sj-docx-2-jnd-10.1177_22143602261419558.docx]

**Supplemental Table 2**. Absolute and change from baseline in average total RSA Q1-Q5 with 500g wrist weight (Full Analysis Set).

|  | **Baseline** | **Week 4** | **Week 12** | **Week 24** | **Week 36** | **Week 48** |
| --- | --- | --- | --- | --- | --- | --- |
| Losmapimod, RSA Mean (SD) | 0.508 (0.170) | 0.522 (0.163) | 0.526 (0.171) | 0.523 (0.172) | 0.520 (0.173) | 0.518 (0.170) |
| Losmapimod, CFB Mean (SD) | - | 0.014 (0.051) | 0.019 (0.062) | 0.017 (0.067) | 0.016 (0.069) | 0.011 (0.070) |
| Losmapimod, %CFB Mean (SD) | - | 4.25 (11.6) | 4.70 (12.9) | 3.81 (14.8) | 3.43 (15.5) | 2.88 (15.6) |
| Losmapimod, n | 130 | 126 | 127 | 123 | 123 | 122 |
| Placebo, Mean (SD) | 0.531 (0.161) | 0.554 (0.165) | 0.560 (0.167) | 0.545 (0.175) | 0.533 (0.176) | 0.536 (0.171) |
| Placebo, CFB Mean (SD) | - | 0.021 (0.051) | 0.026 (0.060) | 0.012 (0.068) | 0.003 (0.071) | 0.005 (0.068) |
| Placebo, %CFB Mean (SD) | - | 5.11 (15.8) | 5.88 (15.9) | 3.10 (18.0) | 0.96 (19.5) | 1.81 (17.3) |
| Placebo, n | 129 | 127 | 126 | 120 | 120 | 121 |

CFB = Change from baseline

n = Number of participants in category/analysis at each timepoint

Q1-Q5 = Quadrant 1 to Quadrant 5

RSA = Relative Surface Area

SD = Standard Deviation

Average = Mean of both arms; baseline = Last non-missing value prior to first dose of study drug

For change from baseline, only participants with both a baseline value and a value at the visit timepoint are included

%CFB = Relative CFB = 100 X (Post-Baseline value - Baseline Value)/(Baseline Value)
